# The role of lactylation in tumor growth and cancer progression

**DOI:** 10.3389/fonc.2025.1516785

**Published:** 2025-02-04

**Authors:** Khulood Al-Malsi, Sinan Xie, Yunshi Cai, Nader Mohammed, Kunlin Xie, Tian Lan, Hong Wu

**Affiliations:** ^1^ Department of General Surgery, West China Hospital, Sichuan University, Chengdu, China; ^2^ Liver Transplant Center, Transplant Center, West China Hospital, Sichuan University, Chengdu, China; ^3^ Laboratory of Hepatic AI Translation, Frontiers Science Center for Disease-Related Molecular Network, West China Hospital, Sichuan University, Chengdu, China; ^4^ Emergency Department Research, Hamad Medical Corporation, Doha, Qatar

**Keywords:** lactylation, tumor microenvironment, cancer metabolism, epigenetic regulation, histone, epigenetic modification

## Abstract

**Background:**

Lactate’s perception of lactate has changed over the last 30 years from a straightforward metabolic byproduct to a complex chemical with important biological activities, such as signal transduction, gluconeogenesis, and mitochondrial respiration. In addition to its metabolic contributions, lactate has far-reaching repercussions. This review highlights the role of lactate in the course of cancer by highlighting lactylation as a unique epigenetic alteration. The purpose of this review is to clarify the functions of lactate in the biology of tumors, with a particular focus on the translational potential of lactylation pathways in cancer diagnosis and treatment approaches.

**Methods:**

This review summarizes research on the relationship between lactate and cancer, with an emphasis on histone lactylation, its effect on gene expression, and its influence on the tumor microenvironment. By establishing a connection between metabolic byproducts and epigenetic gene regulation, we investigated how lactylation affects immune regulation, inflammation, and cellular repair.

**Findings:**

Histone lactylation, or the addition of lactate to lysine residues on histone proteins, increases transcriptional activity and facilitates the expression of genes involved in homeostasis and repair. These findings have important implications for cancer treatment. Lactylation, for example, activates genes such as Arg1, which is a hallmark of the M2 macrophage phenotype implicated in immunosuppression and tumor growth. The ability of lactate to dynamically alter gene expression is further supported by its function as a histone deacetylase(HDAC)inhibitor and its impact on histone acetylation. Its wide-ranging involvement in cellular metabolism and epigenetic control has been demonstrated by the discovery of particular lactylation sites on histones in various cell types, including cancer cells.

## 1 Introduction

Over the past thirty years, an increasing body of research has shown that lactate performs various biological functions. These include acting as signal transduction molecules, converting into glucose in the gluconeogenesis pathway, and converting into pyruvate and adenosine triphosphate (ATP) through mitochondrial respiration complex molecular and cellular processes that promote cancer development and metastasis are involved in tumor progression (1, 2). Post-translational modifications(PTMs) of proteins are essential for controlling their activities. Lactylation is the process by which lactate is added to lysine residues in proteins. It is a unique PTM linked to cancer biology. Lactate is the final byproduct of glycolysis. A growing number of researchers have pointed to lactate as a crucial energy source for mitochondrial respiration and as a precursor to gluconeogenesis, as a result of the introduction of the lactate shuttle idea in the early 1980s. Through receptors expressed in a variety of cells, lactate also functions as a multifunctional signaling molecule with a range of biological effects such as immune regulation, anti-inflammatory wound healing, reduced lipolysis, and improved exercise performance in relation to the gut microbiome (3, 4).Moreover, mounting data indicate that lactate plays a critical role in immunological modulation and homeostasis maintenance by lactylating lysine residues of histones, which in turn contributes to epigenetic gene regulation.

Although lactic acid has long been thought of as a waste product of glycolytic metabolism, two separate groups discovered in 2017 that in both normal tissues and tumors, it can be recycled as the main carbon source for the mitochondrial tricarboxylic acid (TCA) cycle. It was recently shown that lactic acid integrates into cell metabolism by causing dynamic variations in Mg2+ levels between the endoplasmic reticulum and the mitochondria. As a HDAC inhibitor, lactate has also been shown to control gene expression and enhance histone acetylation. Additionally, it was recently shown that lactic acid may alter the lysine residues of histones in a novel epigenetic alteration known as lactylation, which was inspired by the widespread acylation of histones by intracellular metabolites. In human HeLa cells and mouse BMDMs, 28 lysine lactylation (Kla) sites were found on core histones, including H3, H4, H2A, and H2B. Lactylation of H3 and H4 is mediated by p300 and is dependent on p53. Histone lactylation inside cells can be improved by hypoxia and macrophage polarization, which are linked to elevated lactate production by active glycolysis. Increased histone Kla levels directly increase gene transcription and activate homeostatic genes, such as Arg1, a hallmark of M2 macrophages, during the late stages of M1 macrophage polarization. Remarkably, researchers also found histone lactylation in macrophages taken from lung tumors and mouse melanoma, and they noted a positive association between histone lactylation and the ability of reparative M2 macrophages to produce carcinogens (5, 6).

These results imply that excessive amounts of histone lactylation and lactate in macrophages may play a role in the development and spread of tumors (7). It is astonishing how much of an impact one metabolite may have on immune cell activity. Understanding how lactic acid alters other cell types, solving the puzzle of the Warburg effect, and comprehending its implications for human diseases are all made possible by the discovery of histone lactylation and its effects on macrophage biology. It is unknown whether this technique can control immune cells, such as T cells and cancer cells. The Warburg effect has also been linked to sepsis, autoimmune disorders, atherosclerosis, diabetes, aging, and cancer. Further research on the function and control of this recently identified histone alteration is required (3, 4, 8) This review aimed to explore the role of lactylation in tumor progression across different organ systems.

## 2 Introduction to epigenetic modifications and lactylation

Epigenetic modifications represent heritable changes in gene expression that occur without alteration of the underlying DNA sequence. These modifications include DNA methylation, histone modifications, and non-coding RNA regulation, and play a crucial role in regulating chromatin accessibility and transcriptional activity. Epigenetic mechanisms are fundamental to cellular differentiation, development, and disease progression, bridging the gap between genetic predisposition and environmental influence (9–12). Histone modifications, such as acetylation, methylation, phosphorylation, and ubiquitination, are particularly significant because they determine whether chromatin adopts an open (euchromatic) or closed (heterochromatic) conformation, thereby influencing gene expression (12, 13). Recent technological advances, including CRISPR/dCas9-based epigenetic editing, have enabled the precise manipulation of these modifications, offering promising therapeutic avenues (14, 15) A key link between metabolism and epigenetic regulation has also emerged, as metabolites like acetyl-CoA and S-adenosylmethionine (SAM) directly influence histone acetylation and methylation, highlighting the interconnectedness of chromatin dynamics and cellular metabolism (15, 16).

Lactylation is a recently discovered post-translational modification that adds a new dimension to epigenetic regulation. This process involves the addition of lactate-derived moieties to lysine residues and is mediated by lactate, a by-product of glycolysis. Initially identified in macrophages, lactylation has been shown to influence histone and non-histone proteins and regulate key biological processes, such as immune response, tissue repair, and cellular metabolism (5, 17). For example, histone lactylation, including modifications such as H3K18La, plays a role similar to that of acetylation by activating gene transcription. It is particularly significant in highly glycolytic environments where it enhances chromatin accessibility at genomic loci linked to cellular differentiation and metabolic adaptation (18, 19).

## 3 Metabolic-epigenetic link in tumors

**Figure 1:**
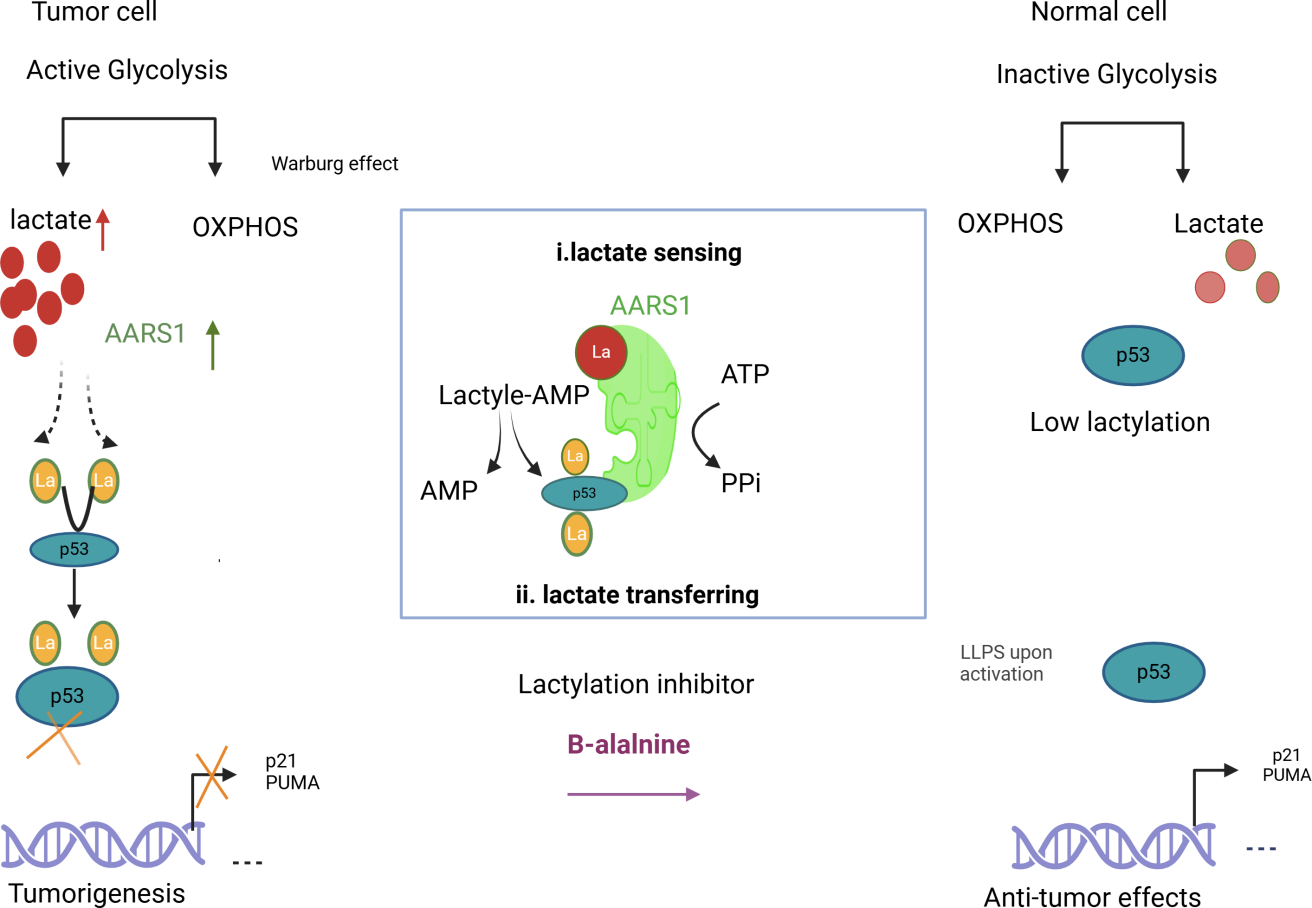
Illustrates how lactylation increases lactate and AARS1 activity, shifting p53 from its anti-tumor role in healthy cells to a tumor-promoting role in cancer cells. It also suggests β-alanine as a potential therapy to target lactylation in cancer treatment.

In tumors, the interplay between metabolism and epigenetic regulation is even more critical. Cancer cells often exhibit metabolic reprogramming, such as the Warburg effect, which leads to increased lactate production. This excess lactate drives lactylation, a modification that reprograms gene expression to promote tumor growth, angiogenesis, immune evasion, and therapy resistance (19–21). Histone lactylation in tumor cells has been shown to activate oncogenic pathways and suppress tumor-suppressive mechanisms. For instance, lactylation can interfere with the function of critical tumor suppressors such as p53 by altering the expression of genes involved in cell cycle arrest and apoptosis (20, 21). Furthermore, non-histone protein lactylation affects transcription factors and DNA repair proteins, such as NBS1 K388, enhancing tumor cell survival and resistance to therapeutic interventions (21). Emerging evidence highlights that lactylation is a key mechanism by which metabolic states influence chromatin dynamics, aligning gene expression with cellular metabolic requirements. This metabolic-epigenetic link underscores the dual role of lactylation in normal and tumor cells, offering potential therapeutic opportunities for targeting lactylation to restore tumor-suppressive functions (16, 22, 23) (**Figure 1**).

## 4 Lactylation and cellular physiology

It has been demonstrated that lactylation, which was previously only known to occur during metabolism, has a variety of consequences on cellular physiology. In addition to its roles in metabolism, lactylation affects the stability, activity, and interactions of proteins, which in turn affect several cellular processes, such as signal transduction, cytoskeletal dynamics, and gene expression. Plasma lactate concentration is automatically determined by blood gas analysis and is normally between 0.3 and 1.3 mmol litre-1. This indicates an equilibrium between lactate synthesis and metabolism. Humans produce the levorotatory isoform of lactate. The skin, skeletal myocytes, perivenous hepatocytes, and erythrocytes normally produce lactate. Spectrophotometric analyzers use nicotinamide adenine dinucleotide (NADH) and lactate to detect lactate in deproteinized blood. Lactate oxidase is incorporated into a modified amperometric cell in blood gas analyzers to convert lactate into hydrogen peroxide (7, 24).Stabilize specimens before analysis; *in vitro* cell glycolysis can cause a misleading increase in whole blood lactate (2, 6). Lactate and lactic acidosis are associated with oxidative phosphorylation, which produces adenosine triphosphate (ATP). Acidosis is caused by the net accumulation of H+, which results from oxidative phosphorylation during lactate formation. 70% of lactate is eliminated by the liver, and diffusion and monocarboxylate transporter are both necessary for proper lactate metabolism.

NAD+, which is partially generated by the conversion of pyruvate to lactate, is necessary for glycolysis. The rate at which pyruvate transforms into lactate is determined by NADH availability. Pyruvate must be converted to acetyl CoA in tissues such as the heart. To convert NADH back into NAD+, shuttles were used to move the electrons across the mitochondrial membrane (24, 25). Concentrations of NADH increase and lactate synthesis regenerates NAD+, elevating lactate concentrations, if the rate of glycolysis increases to the point where the ox-phos shuttle is overloaded. Errors in metabolism, decreased lactate clearance, or elevated lactate generation can lead to hyperlactemia. NAD+ is required for increased glycolysis, which occurs when pyruvate is converted to lactate. Since phosphofructokinase (PFK) activity is rate-limiting, conditions such as hypoxemia, anemia, hypoperfusion, extreme exercise, and carbon monoxide poisoning cause a drop in ATP, which in turn stimulates PFK as AMP rises. Endotoxin and thiamine deficiencies are examples of metabolic errors that can lead to pyruvate buildup. Hyperlactemia can also result from decreased elimination of hepatic lactate (5, 6, 8, 24, 25). Endotoxin and thiamine deficiencies are examples of metabolic errors that can lead to pyruvate buildup. Hyperlactemia can also result from decreased elimination of hepatic lactate.

Therefore, lactate serves an important function as a primary energy source, gluconeogenic precursor, and signaling molecule, in addition to being a waste product of anaerobic metabolism. It functions by mass action, controlling lipid oxidation and energy partitioning, as well as cell redox, ROS production, and histone lactylation. The exchange of lactate between cells facilitates metabolic flexibility and energy equilibrium, and repeated exposure triggers adaptive processes, such as mitochondrial biogenesis. The mitochondrial enzyme lactate oxidase can produce H2O2, which affects the generation of reactive oxygen species and wound healing (7, 26).

**Figure 2:**
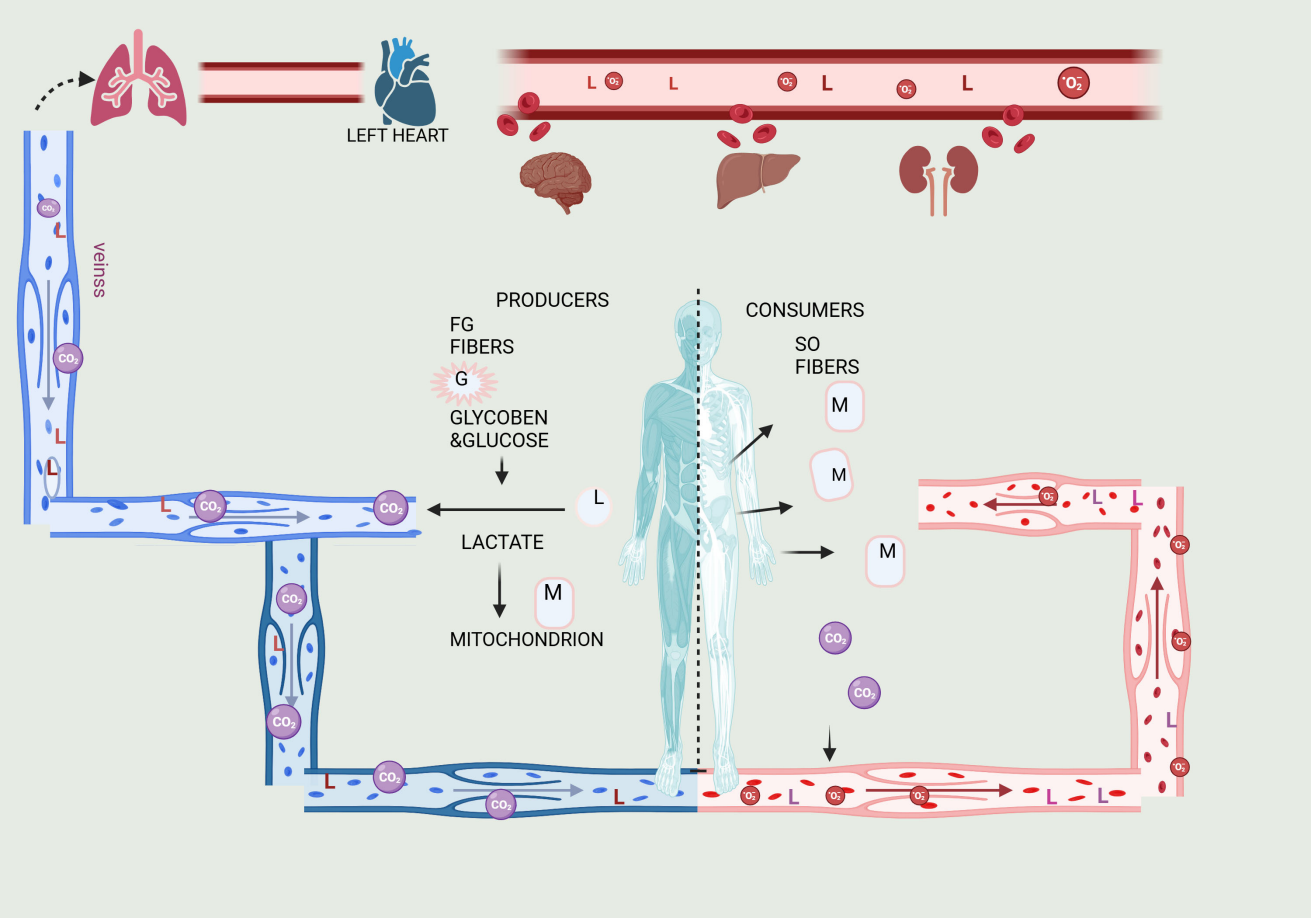
Illustration of the Lactate Shuttle, explaining lactate’s roles in cell signaling and the transport of oxidative and gluconeogenic substrates. Lactate transfers occur between producer (or driver) white-glycolytic (FW) and red-oxidative (SO) consumer (or receiver) fibers within a working muscle bed, as well as between producer working skeletal muscle and consumer organs such as the heart, brain, liver, and kidneys. These interactions exemplify cell-cell lactate shuttles. Additionally, intracellular lactate shuttles occur between the cytosol and peroxisomes, and between the cytosol and mitochondria. Most, if not all, lactate shuttles are driven by redox states, concentration gradients, or both. The letters G represent glucose and glycogen, L represents lactate, and M represents mitochondrial reticulum components.

**Figure 3:**
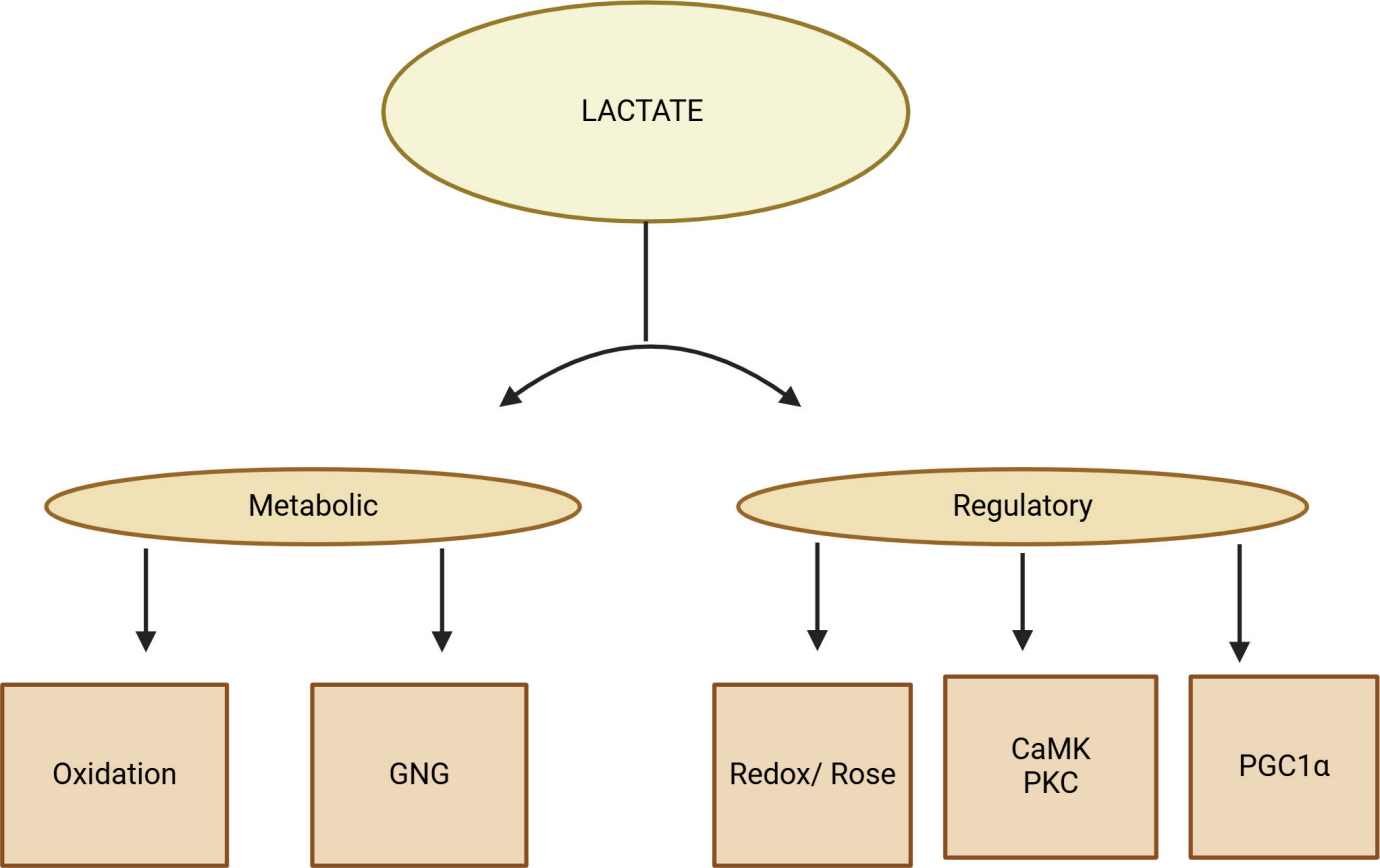
A diagram displaying the lactate shuttle performing three physiological functions. Due to its autocrine, paracrine, and endocrine-like properties, lactate acts as a primary energy source, a major gluconeogenic precursor, and a signaling molecule, known as a “lactone.” These functions can be categorized as metabolic (oxidative fuel) and gluconeogenic (GNG) activities, as well as regulatory or signaling functions.

Lactate can control the expression of genes by lactylating histones, which can alter chromatin structure and potentially impact the production of metabolic proteins under different circumstances. Diseases, such as cancer, can cause dysregulation of the lactate signaling pathway (**Figures 2**, **3**).

## 5 Lactylation in tumor microenvironment

### 5.1 Lactylation of tumor cells in TME

In addition to being the product of glycolysis, lactate is also gradually being shown to serve as a universal metabolic fuel for energy through the movement of molecules between cells called the “lactate shuttle.” Lactate plays a key role in energy metabolism reprogramming, allowing cells to receive copious amounts of energy in a short amount of time. This is due to glycolytic-dependent metabolism that is prevalent in tumors and fast-growing cells. Furthermore, lactate can promote tumorigenesis by influencing the acidic tumor microenvironment and attracting immune cells. The discovery of lactate-induced lactylation has recently shed more light on the mechanisms underlying lactate production, circulation, and utilization that promote tumorigenesis (26–28). Lactylation can modify histone proteins to alter the spatial arrangement of chromatin, impact DNA accessibility, and control the expression of related genes, similar to other epigenetic alterations. Furthermore, the spatialized lactate concentration and degree of lactylation are inextricably linked, establishing a connection between metabolic reprogramming and epigenetics. Here, we discuss the significance of lactate in energy reprogramming, discuss the most recent discovery regarding the role of lactylation in carcinogenesis, and attempt to investigate dual-targeting monotherapy treatment approaches (24, 27, 28).

**Figure 4:**
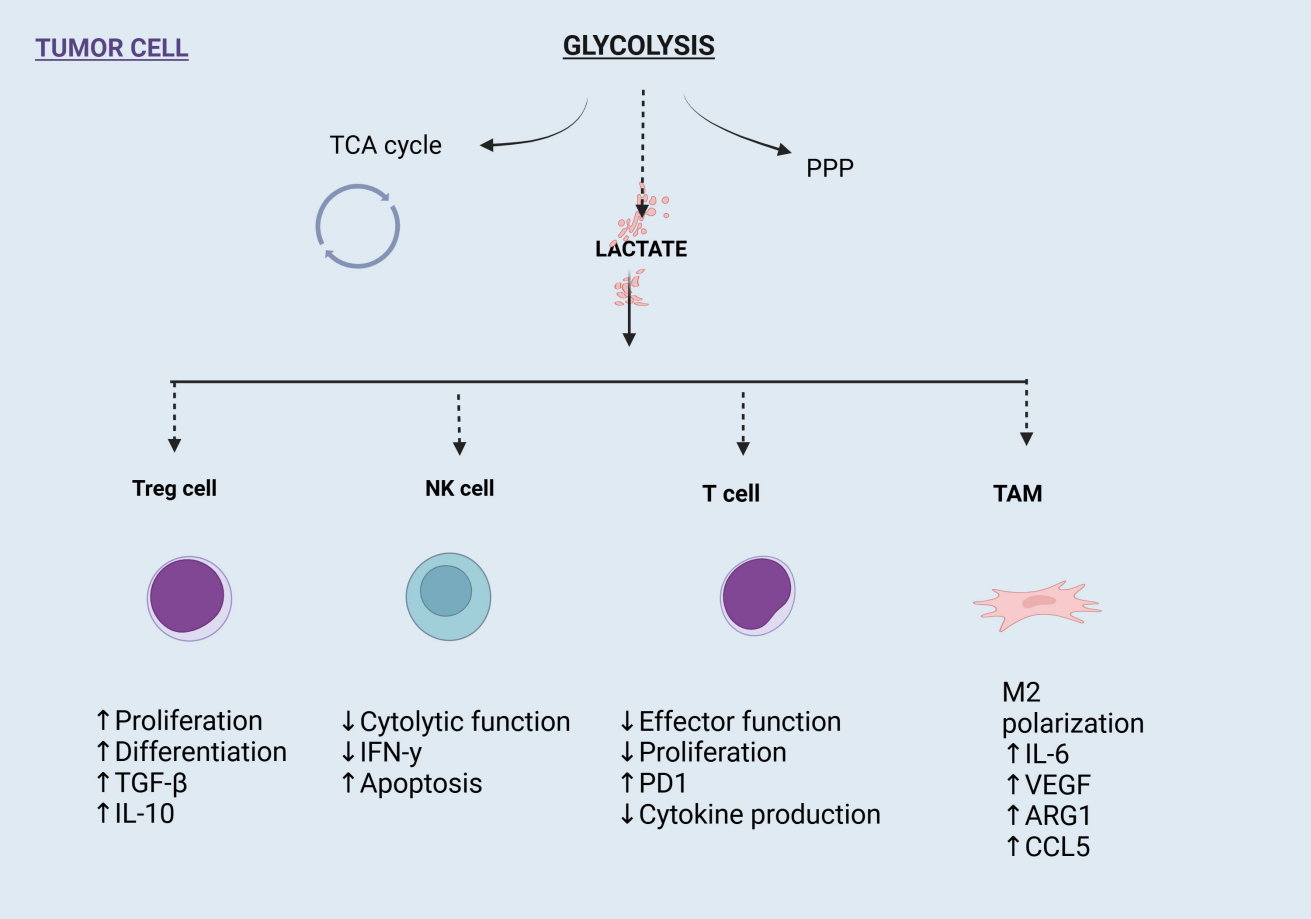
Impact of glycolysis-derived lactate on immune cells in the tumor microenvironment (TME). Lactate enhances Treg cell proliferation and immunosuppressive cytokines, suppresses NK and T cell functions, and drives M2 polarization of tumor-associated macrophages (TAMs), promoting tumor progression and immune evasion.

In the tumor microenvironment, lactate is an essential substrate for energy metabolism, which promotes both energy metabolism and epigenetic alterations. It is carried by a “lactate shuttle,” which modifies the amount of lactate present in the cells and surroundings. In the tumor microenvironment, a number of important lactylation sites are present in immune cells, which alter the milieu and encourage the development and spread of tumors. As targeted lactate production can enhance the tumor microenvironment and prevent or improve lactylation modification, its investigation as a cancer treatment technique seems encouraging (2, 26, 27). Because lactate-induced lactylation shapes the tumor microenvironment and facilitates tumor survival and progression, it is a key factor in tumor development. To alter the tumor microenvironment, it can draw in and control cancer-associated factors (CAFs), tumor-infiltrating myeloid cells (TIMs), and cancer stem cells (CSCs). By inducing VEGF expression and TAM polarization to an M2-like phenotype, lactate stimulates tumor growth. Arg 1 and VEGF are expressed more frequently when macrophages are activated by tumor-derived lactate signaling through HIF1α. Additionally, this mechanism promotes the growth of blood vessels and inflammation. Atherosclerosis has been linked to this lactylation-mediated shift from pro- to anti-inflammatory macrophages (2, 24, 26, 27) (**Figure 4**).

### 5.2 Lactylation of tumor cells and tumor progression

**Figure 5:**
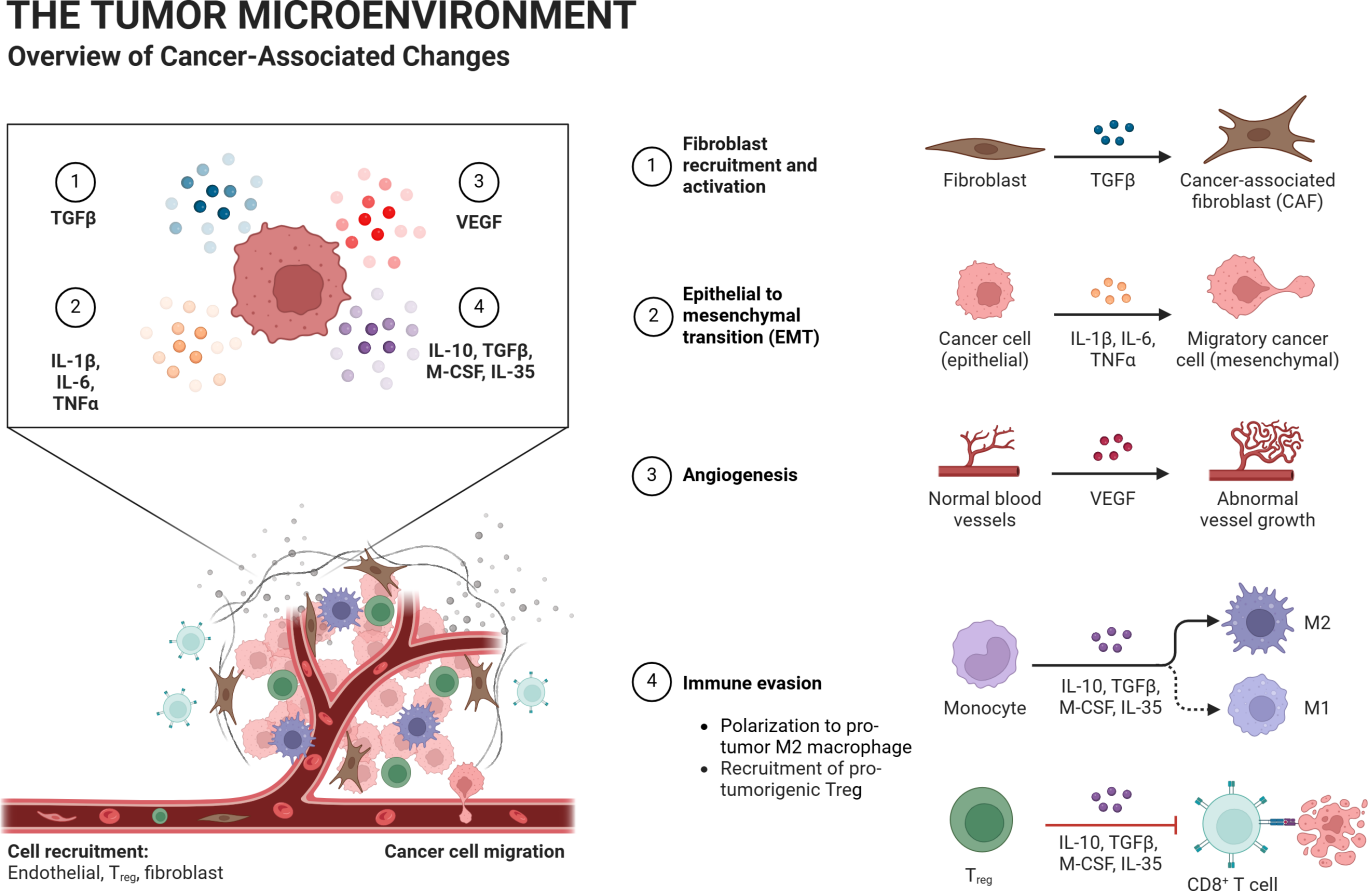
The tumor microenvironment consists of tumor cells, tumor-associated immune cells, and tumor-associated fibroblasts. Lactate and lactylation regulate this environment. The hypoxic regions of the tumor microenvironment often have the highest lactate levels, with cell metabolism relying heavily on glycolysis. As oxygen supply increases, the lactate concentration gradient decreases. Lactate produced by “lactate-producing cells” diffuses to neighboring cells, enabling further conversion of the borrowed lactate within those cells.

Lactylation, one of the main components of TIMs, is essential for immunological tolerance maintenance and tumor immune escape. The ability of TIMs to inhibit the immune system is strengthened via the METTL3/m6A/JAK1/STAT3 axis. In the tumor microenvironment, regulatory T (Treg) cells also sustain an immunosuppressive state. MOESIN lactylation at Lys affects the production of Treg cells and increases TGF-β signaling. The antitumor efficacy of combination therapy combining anti-PD-1 and lactate dehydrogenase inhibitors is greater than that of anti-PD-1 medication alone. Additionally, lactylation controls cell pluripotency, which initiates the tumorigenesis-related epigenome-metabolome-epigenome cascade. Lactylation signals are concentrated in the YTHDF2 promoter region in ocular melanoma, which has been found to function as an oncogene in a variety of tumors. By encouraging oncogenesis, lactylation in tumor tissues can have a detrimental effect on the prognosis of ocular melanoma (5). It promotes cancer and metastasis by directly controlling gene expression. In colorectal cancer and clear cell renal cell carcinoma (ccRCC), lactylation is increased, which stimulates PDGFRβ signaling to advance the tumor. It is also essential in non-small cell lung cancer (NSCLC) and hepatocellular carcinoma (HCC) (8, 24–26) (**Figure 5**; **Table 1**).

**Table 1:** Locations and roles of lactylation modifications in various cell types (5, 6, 27).

Cells	Lactylation Sites	Function
ATC cell	H4K12	Gene transcription and cell cycle dysregulation and ATC deterioration
MCF-7	H3K9,18,23,27,56,122 H4K5,8,12,31,77,91	N/A
BMDM	H3K14,18,23,27,56	Tumor cell proliferation
LLC1	H3K18	N/A
B16F10	H3K18	N/A
HEK293T	H3K18	N/A
HCT116	H3K18	N/A
HeLa	H3K9,18,23,27,79 H4K5,8,12,16,31,77,91 H2AK11,13,115 H2BK5,11,15,16,20,23,43,85,108,116,120	N/A
Ocular melanoma cells	H3K18	PER1 and TP53 mRNAs degradation and ocular melanoma progression
ccRCC cells	H3K18	PDGFRb transcription activation and ccRCC progression
HCC cells	K28; H3K18	AK2 inhibition and HCC proliferation; N/A
LCSCs	H3K9, H3K56	HCC proliferation
Microglia	K183	Increased expression of FGF2 and Proliferative Retinopathy progression
TIMs	H3K18	METTL3 upregulation and CRC promotion
Tregs	K72	Enhancing TGF-b signaling, efficient Tregs production and HCC progression.

N/A, Not Applicable.

## 6 Organ-specific roles of lactylation in tumor

The role of lactylation, a recently discovered post-translational modification, in tumor progression varies across different organs and influences distinct aspects of cancer development and metastasis. This section discusses the organ-specific roles of lactylation in driving tumor progression.

### 6.1 Lactylation in primary liver cancer

Hepatocellular carcinoma (HCC) is one of the most frequent cancer diagnoses and the leading cause of cancer-related mortality globally. It is a highly heterogeneous malignant tumor with considerable histological and metabolic variabilities. Currently, available treatments mostly consist of radiation, immunotherapy, chemotherapy, surgery, and interventional therapy. Despite the effectiveness of several medicines and different diagnostic approaches, the 5-year overall survival (OS) rate of patients with head and neck cancer remains below 50%.

Hepatocellular carcinoma (HCC) is a common cancer. HCC spreads and multiplies because of lactylation. The objective of this study was to create a novel scoring system based on lactylation-related genes (LRGs) for prognosticating HCC, immunotherapy, single-cell sequencing, and treatment sensitivity, although lactylation has not been extensively studied in the construction of prognostic markers for HCC. Hepatocellular carcinoma (HCC) is a common cancer. HCC spreads and multiplies because of lactylation. The objective of this study was to create a novel scoring system based on lactylation-related genes (LRGs) for prognosticating HCC, immunotherapy, single-cell sequencing, and treatment sensitivity, although lactylation has not been extensively studied in the construction of prognostic markers for HCC.A growing field of study in oncology is the function of lactylation in primary liver cancer, specifically in hepatocellular carcinoma (HCC). The insertion of lactate to lysine residues in proteins is a post-translational alteration that affects gene regulation, cellular metabolism, and interactions between the tumor and its surroundings. These processes are critical for the onset and spread of liver cancers.

### 6.2 Role of lactylation in HCC

proteins had a significant impact on gene expression and cellular metabolism. This alteration is especially important in HCC because of the Warburg effect, a change in glycolytic activity that causes cancer cells to produce excessive lactate. This extra lactate is a metabolic waste product that plays a vital regulatory role in the tumor microenvironment, impacting immune and tumor cells alike.

Lactylation is a regulatory mechanism connecting metabolic conditions to epigenetic modifications. Lactylation affects many cellular functions in HCC, and metabolic alterations are a defining feature. These functions include invasion, proliferation, and adaptation to hypoxic conditions. It stabilizes hypoxia-inducible factors and other transcriptional activators, increasing cancer cell survival under metabolic stress.

#### 6.2.1 Demethylzeylasteral and histone lactylation

Lactate is the target of demethylzeylasteral, which inhibits the tumorigenicity of liver cancer stem cells. Demethylzeylasteral reduces the tumorigenicity of liver cancer stem cells by blocking histone lactylation, which is a critical step in tumor formation, and inhibits glycolysis by lowering lactate synthesis. Reduced tumorigenicity in liver cancer stem cells (LCSCs) is linked to DML’s inhibition of glycolysis and the targeting of histone H3 lactylation, particularly at H3K9 and H3K56. Altering lactylation provides a novel treatment avenue, as supported by additional research and omics analyses concentrating on histone and non-histone proteins. Subsequent investigations utilizing cutting-edge methodologies such as LC-MS/MS and CHIP-seq/RNA-seq will delve deeper into molecular pathways to augment our understanding and management of hepatocellular carcinoma. Studies on the effects of demethylzeylasteral (DML) on liver cancer stem cells (LCSCs) shown that DML targets histone lactylation and lactate production to reduce the tumorigenicity of these cells. One post-translational change that increases cancer cell aggressiveness is histone lactylation. It is controlled by cellular metabolism, particularly by glycolysis (29, 30).

According to previous studies, DML reduces the amount of lactate produced, which is an important metabolite in glycolysis, and results in less histone lactylation. The lower tumorigenic potential of LCSCs is associated with a reduction in histone lactylation. This process entails altering important molecular pathways, such as those affecting cell survival and proliferation, that control the fate of cells in liver cancer. As DML targets the metabolic and epigenetic landscapes of cancer stem cells, its effects on these cellular processes suggest that it may be used as a therapeutic agent to treat liver cancer (29, 30).

#### 6.2.2 In-depth discovery of protein lactylation in hepatocellular carcinoma

Thorough analysis of protein lactylation in HCC sheds light on the potential use of lactylation as a target for therapeutic intervention, and offers important insights into the molecular mechanisms underlying the development of cancer. The work found 2,045 Kla modification sites on 960 proteins using sophisticated proteomic techniques, including antibody-based Kla-peptide enrichment and high-throughput liquid chromatography-tandem mass spectrometry (LC-MS/MS). Interestingly, 1,438 of these locations from 772 proteins were quantitatively examined(17). The results showed the notable presence of Kla proteins with variable expression, which may play a role in the development and metastasis of HCC. Certain Kla sites in proteins, including ATP-binding cassette family 1 (ABCF1) and ubiquitin-specific peptidase 14 (USP14), have been identified as putative indicators of HCC and its spread. These proteins may be targets for treatment and diagnostics because they are involved in important biological processes related to tumors. By demonstrating the broad role that lactylation plays in controlling tumor biology, this work represents a major advancement in the field of cancer proteomics and creates new avenues for the creation of lactylation-based diagnostics and treatments (31) (**Table 2**).

**Table 2:** Provides an overview of the research accomplishments and their potential impact on HCC diagnosis and therapy (35, 62).

Finding	Implication	Protein Examples
Finding the lactylation sites	960 proteins including 2,045 lactylation sites were found, providing a thorough map of lactylation in HCC.	USP14, ABCF1
proteins with varying expression	identified proteins with particular lactylation patterns that may be used as indicators to anticipate metastases and diagnose HCC.	USP14, ABCF1
Diagnostic	Specific lactylation sites on USP14 and ABCF1 could serve as diagnostic markers for HCC and its metastasis.	USP14 (K336), ABCF1 (K430)
implications for therapy	emphasized the possibility of creating tailored treatments to treat HCC by preventing lactylation.	

#### 6.2.3 The role of lactylation in HCC progression under hypoxia

Lactylation is a key factor that drives the formation and progression of hepatocellular carcinoma (HCC) in a hypoxic environment. This work emphasizes the connection between lactate generation and the glycolytic enzyme Aldolase A (ALDOA), showing that lactate not only serves as a metabolic fuel, but also participates in epigenetic control by lactylating histone lysine residues. This alteration affects the expression of genes essential for preserving macrophage homeostasis inside the tumor microenvironment (TME). According to this study, the TME’s high lactate concentrations from tumor cells aid in the recruitment of myeloid-derived suppressor cells, which in turn promotes immunosuppression and tumor growth and migration under hypoxic conditions (32). This complex role of lactate, especially its contribution to histone lactylation, represents a turning point in our understanding of how metabolic byproducts can affect immunological responses and epigenetic changes in the TME, ultimately driving the spread and metastasis of cancer. It summarized in **Table 3** (32).

**Table 3:** Highlights the significant influence of lactylation on the clinical landscape of HCC and its potential to identify new therapeutic targets (35).

Category	Details
Differentially Lactylated Proteins (DLPs)	164 DLPs and 200 modification sites upregulated in HCC vs. normal liver tissues; 8 proteins and 11 modification sites downregulated.
Functional Enrichment in HCC vs Normal Liver.	DLPs are involved in crucial biological processes like amino acid metabolism, ribosomal protein synthesis, and fatty acid metabolism.
Protein-Protein Interaction (PPI) Networks	PPI analysis showed 111 nodes with 107 upregulated and four downregulated proteins in HCC compared to normal liver tissues
Significant Proteins and Modifications	USP14 and ABCF1 identified as significant proteins with altered lactylation, which are involved in tumor metabolism and are potential targets for further research
Subcellular Localization of DLPs.	Mainly localized in the cytoplasm, indicating the central role of lactylation in cellular metabolic processes in cancer cells.
KEGG Pathway Enrichment	Notch and AMPK pathways were identified among others, suggesting an involvement of lactylation in key signaling pathways influencing cell fate and energy metabolism
Future Research Directions	Further investigation on the role of specific lactylated proteins like USP14 and ABCF1 in HCC progression and potential therapeutic targeting

#### 6.2.4 Exploring the prognostic role of histone lysine lactylation-related gene signatures in hepatocellular carcinoma

A newly discovered post-translational alteration that is becoming increasingly significant in the study of cancer biology is histone lysine lactylation (Kla). Together, the two investigations by Wu et al. (2023) and Cheng et al. (2023) add to our knowledge of the crucial role of histone lysine lactylation (Kla) in the development of hepatocellular carcinoma (HCC). Their results highlight the influence of gene signatures associated with lactylation on prognosis and response to therapy (33, 34). Kla is essential for immunological evasion, metabolic adaptability, and tumor development. New opportunities for HCC treatment options in the future have been presented through the discovery of NR6A1, OSBP2, and UNC119B as therapeutic targets. The incorporation of lactylation-associated gene signatures into patient stratification may enhance the process of selecting individualized medications, thereby augmenting the effectiveness of targeted therapies and chemotherapy (33, 34).

These combined findings provide compelling evidence in favor of further investigation into the molecular processes underlying Kla and the creation of novel treatment approaches that focus on lactylation-related pathways. Clinicians and researchers can develop precision oncology in hepatocellular carcinoma and improve patient outcomes by utilizing these discoveries. Together, our findings demonstrate the important role of histone lysine lactylation in determining tumor metabolism and treatment resistance in hepatocellular carcinoma. Together with a predicted lactylation-related gene profile, the discovery of important therapeutic targets, including NR6A1, OSBP2, and UNC119B, presents a novel approach for patient classification and individualized care. Enhancing the efficiency of chemotherapy and the response rates to immunotherapy in HCC may be possible by focusing on lactylation pathways (33, 34).

#### 6.2.5 Comprehensive profiling of protein lysine lactylation in hepatocellular carcinoma insights and implications

Hong et al. completed a revolutionary proteomics study published in April 2023. This study involved a thorough examination of lysine lactylation in hepatocellular carcinoma (HCC). This work, which originated from the Department of General Surgery at the First Affiliated Hospital of Soochow University in Suzhou, Jiangsu Province, China, significantly advanced our knowledge of epigenetic changes linked to cancer growth (35). In addition to improving our understanding of tumor biology, researchers’ comprehensive description of the ubiquitous role of lysine lactylation in changing protein function in HCC opens up new possibilities for treatment approaches. The results highlight the intricate nature of post-translational alterations in cancer and indicate that novel approaches to treating head and neck cancer (HCC) may be possible through focused alteration of lactylation pathways. Furthermore, promising targets for future medication development are highlighted by the discovery of variably lactylated proteins implicated in crucial biological processes, such as fatty acid metabolism, ribosomal protein synthesis, and amino acid metabolism. This work offers a strong basis for further investigations into the processes of lactylation in HCC, which might lead to improved therapeutic strategies and better results for patients with this difficult cancer (35). The comprehensive proteome analysis carried out in the earlier research is summarized in **Table 3**.

Yang et al. investigated the role of lactylation in metabolic reprogramming and cancer progression by focusing on the lactylome of HCC tissues from patients infected with the hepatitis B virus (HBV). Among their most important achievements are 2,497 proteins have been found to have extensive lactylation, which impacts important metabolic processes and enzymes. There is a correlation between poor clinical outcomes and increased tumor development at particular lactylation sites, including K28 on adenylate kinase 2, which may be directly related to promotion of cancer cell proliferation and metastasis. This study focused on how lactylation affects the metabolic landscape of HCC, especially when it comes to controlling pathways, such as the tricarboxylic acid cycle and glycolysis. The findings of this study on the involvement of lactylation in enzymatic and metabolic modulation that promotes HCC progression provide new opportunities for targeted therapy. The therapeutic potential of lactylation modulation warrants further investigation, as it may result in more efficacious treatments for HCC, especially in cases associated with HBV (36). **Table 4** provides a thorough summary by contrasting and comparing the important contributions made by both researchers to our knowledge of lysine lactylation in hepatocellular cancer.

**Table 4:** Provides a thorough summary, comparing and contrasting the key contributions made by both researchers to our understanding of lysine lactylation in hepatocellular cancer.

Category	Hong et al., 2023	Yang et al., 2023
Study Focus	Protein lysine lactylation profiling in lung metastasis, normal liver, and HCC tissues	Examining the function of lysine lactylation in HBV patients’ metabolic reprogramming and HCC development
Methodology	Finding and measuring lactylation modification sites by extensive proteome analysis	Analysis of proteomic lactylome with a focus on HCC tissues
Key Findings	2,045 lactylation sites were found in 960 different proteins.- Notable alterations in the proteins involved in metabolic pathways- USP14 and ABCF1 as possible targets	A significant amount of lactylation in 2,497 proteins- Adenylate kinase 2 specific sites, such as K28, are connected to the advancement of cancer
Implications for the metabolism of cancer	Highlighted how lactylation affects metabolic enzymes that are essential to pathways influencing the growth and spread of tumors.	Shown that lactylation affects important metabolic pathways, promoting the spread and proliferation of HCC cells.
Potential Therapeutic Targets	Due to their involvement in tumor metastasis and progression, ABCF1 and USP14 were discovered.	Lactylation sites that may be targeted in order to interfere with the tricarboxylic acid cycle and glycolysis, two major HCC metabolic pathways
Prognostic Value	Suggested that distinct lactylation patterns could function as biomarkers for the development of tumors and the potential for metastasis	Highlighted how important lactylation patterns are as a predictive factor for predicting clinical outcomes.
Therapeutic Implications	suggested using targeted lactylated proteins in treatments to prevent the development of HCC	pushed for the creation of therapies that alter lactylation to prevent cancer cells from undergoing metabolic changes

Each study offers a unique perspective on how this post-translational modification could be leveraged to improve HCC diagnostic and treatment strategies (35, 36).

#### 6.2.6 mcPGK1-dependent mitochondrial import of PGK1 promotes metabolic reprogramming and self-renewal of liver TICs

According to a previous study, mcPGK1, a circular RNA encoded by the mitochondria, plays a critical role in changing the metabolic balance in liver TICs from oxidative phosphorylation (OXPHOS) to glycolysis. The ability of liver TICs to self-renew and develop into tumors is dependent on this metabolic change, which also increases the resistance of tumors to traditional treatments and makes hepatocellular carcinoma more aggressive (37). Moreover, this study highlights the involvement of mcPGK1 in regulating intracellular concentrations of important metabolites such as lactate and α-ketoglutarate, which impact vital signaling pathways such as Wnt/β-catenin, which are essential for the survival and function of liver TIC. The results of this investigation not only broaden our knowledge of the metabolic intricacies linked to the advancement of liver cancer but also point to possible novel targets for therapeutic intervention targeting the metabolic needs of cancer stem cells. The study’s ramifications go beyond liver cancer; it provides information about the metabolic reprogramming common to several forms of cancer and establishes a framework for further research on the functions of mitochondria in cancer biology. Examination of the significance of lactylation in liver cancer emphasizes the importance of the stability of β-catenin protein and its regulatory mechanisms in liver tumor-initiating cells (TICs). The direct effects of lactic acid on histone lactylation at the promoter of lnc-β-catm facilitate the production of lnc-β-catm, which is critical for β-catenin stability. This process highlights an important epigenetic mechanism by which lactylation directly upregulates the expression of genes linked to important pathways in liver tumor-inducing cells (TICs), thereby accelerating the evolution of liver cancer and TIC self-renewal (37).

#### 6.2.7 Role of CENPA lactylation in hepatocellular carcinoma progression

Recent developments in our knowledge of post-translational changes, such as lactylation, have illuminated the novel pathways underlying the development of tumors. Liao et al. examined the function of centromere protein A (CENPA) in the development of hepatocellular carcinoma (HCC). According to this study, HCC tissues had far higher levels of CENPA than nearby non-tumor tissues. The researchers discovered that lysine lactylation at K124 increases CENPA’s transcriptional activity of CENPA, which in turn promotes the production of important oncogenes, such as neuropilin 2 (NRP2) and cyclin D1 (CCND1). Moreover, CENPA and the transcription factor YY1 work together to create a transcriptional complex that controls the expression of CCND1 and NRP2, promoting the growth and metastasis of HCC. While overexpression of CENPA increases target gene transcription and accelerates cell proliferation, CENPA knockdown inhibits tumor development. The carcinogenic potential of CENPA is greatly increased by lactylation at position K124, highlighting the critical role of this post-translational modification (PTM) in controlling tumor behavior. According to their findings, CENPA is lactylated at lysine 124, dramatically increasing its transcriptional activity. Due to this alteration, CENPA can work with the transcription factor YY1 to produce a complex that upregulates important oncogenes, such as neuropilin 2 (NRP2) and cyclin D1 (CCND1). These genes are essential for the growth and spread of HCC cells. The authors observed that overexpression of CENPA increased proliferation and colony formation, thereby enhancing the oncogenic effect of this protein, and that CENPA knockdown dramatically suppressed tumor development. Notably, mutations at K124 diminish CENPA’s capacity of CENPA for transcriptional activation, indicating that lactylation plays a critical role in its operation. The study concluded that new treatment approaches for HCC may be provided by focusing on the lactylation of CENPA or the CENPA-YY1-CCND1/NRP2 axis. Tumor development and progression may be inhibited by this axis. Consequently, the discovery of this route offers intriguing new targets for future therapeutic advancement in the battle against HCC (38).

#### 6.2.8 Implications of lactylation in hepatocellular carcinoma - insights from recent studies

HCC is a difficult-to-treat malignancy with a poor prognosis that is frequently associated with HBV infection. Lactylation of unknown post-translational modifications is a crucial element in the metabolism and development of cancer. Yang et al. and Liu et al. provided novel treatment options by highlighting the significance of lactylation in HCC. Yang et al. conducted a thorough lactylome study to investigate non-histone lysine lactylation in HBV-related HCC. They discovered that higher levels of lactylation, which mainly affect metabolic pathways and RNA processing, are associated with aggressive tumor characteristics and poorer outcomes. They conducted a thorough lactylome study that explored non-histone lysine lactylation in relation to HCC caused by hepatitis B virus (HBV). Their results showed that by altering metabolic pathways and RNA processing, higher lactylation is associated with an aggressive tumor phenotype and poor prognosis. This study highlights the important role that lactylation plays in metabolic reprogramming and carcinogenic activity of HCC, indicating potential therapeutic advantages in addressing lactylation (36). Liu et al. developed a predictive model based on five important lactylation-related genes and identified lactylation-related subgroups within HCC. This model provides a new tool for personalized medicine for HCC by predicting responses to immunotherapy and other therapies. They employed five essential genes to construct a predictive model by identifying and analyzing 332 lactylation-related genes (LRGs). This approach successfully differentiated between patients with high- and low-risk HCC. Significant variations in immune cell infiltration and tumor microenvironment between the two groups were highlighted by the linked five-gene risk profile, providing a basis for individualized treatment strategies. Furthermore, Liu et al. developed a thorough nomogram that reliably forecasts patient survival at 1-, 3-, and 5-year intervals by fusing clinical data with a lactylation risk model (39).

The potential use of lactylation indicators in prognostic models and therapeutic approaches has been demonstrated in both studies. The five-gene risk profile proposed by Liu et al. and the metabolic implications investigated by Yang et al. offer a comprehensive understanding of how lactylation promotes cancer development and can be targeted for therapeutic treatments. Patient outcomes can be improved by improving prognosis and therapy customization, which is supported by the incorporation of lactylation data into clinical models (36, 39).

#### 6.2.9 The role of tumor-resident microbiota in colorectal cancer liver metastasis via lactylation and immune modulation

The high rate of metastasis of colorectal cancer (CRC), particularly in the liver, poses a significant clinical challenge. Lu et al. investigated the role of intratumoral microbiota, specifically Escherichia coli, in colorectal liver metastasis (CRLM) and highlighted its impact on lactate production, macrophage polarization, and the immunological landscape of the tumor microenvironment (TME) (40). Their study revealed that E. coli in the TME increases lactate production, which inhibits binding of the NF-κB gene via RIG-I lactylation. This alteration in gene expression suppresses Nlrp3 transcription in macrophages, reducing their tumoricidal activity and fostering an immune-evading environment that is conducive to metastasis. Furthermore, RIG-I lactylation alters the macrophage metabolic and inflammatory pathways, encouraging a shift to an M2-like, pro-tumorigenic state. The immunological response is further compromised as the anticancer activities of regulatory and CD8+ T cells, which are crucial for tumor suppression, are diminished.

The study also identified significant therapeutic implications, such as the potential of a small-molecule RIG-I lactylation inhibitor. This inhibitor not only counteracts M2 macrophage polarization but also increases the sensitivity of CRLM to standard treatments such as 5-fluorouracil, providing a promising new avenue for treatment. These findings underscore the mechanisms by which tumor-resident bacteria contribute to liver metastasis in CRC, revealing the dual role of microbiota in driving lactate-mediated immunosuppression and tumor progression. Moreover, identification of RIG-I as a target for lactylation inhibition presents an innovative therapeutic approach, offering the potential to augment current chemotherapy regimens and improve CRLM outcomes (40).

#### 6.2.10 Role of lactate in enhancing non-canonical pyroptosis and exacerbating liver injury

Acetaminophen-induced liver injury (AILI) is a serious clinical problem that frequently results in failure or severe liver damage. Although the metabolic conversion of APAP is widely known, the underlying pathways that lead to inflammation and subsequent harm are unclear. Recent studies have highlighted the possible involvement of lactate in these processes. A team under the direction of Dr. Qinglin Li set out a pioneering expedition into the depths of liver disease processes at the busy research labs of Xi’an Jiaotong University. The focus was on lactate, an often-overlooked molecule that became a crucial characteristic in the drama of liver damage rather than just a result of cellular activity (41). This study delves into the noteworthy function of lactate in augmenting non-canonical pyroptosis mediated by Caspase-11 in the context of liver damage induced by acetaminophen. Lactate is mostly produced by the metabolic activity of the wounded liver. It raises Caspase-11 levels and helps activate gasdermin D, which in turn promotes macrophage pyroptosis. Liver damage is exacerbated by the pyroptotic pathway. Through a reduction in its association with NEDD4, which typically reduces Caspase-11 levels, lactate mechanistically inhibits the ubiquitination of Caspase-11. A unique regulatory mechanism in liver disease was suggested by the study, which also found a distinct modification in the lactylation of NEDD4 by lactate, which further affected the functional dynamics between NEDD4 and Caspase-11. Lactate plays two roles in inflammatory processes: it is a metabolic byproduct and a regulatory molecule. This interaction sheds light on the multiple roles of lactate and suggests potential treatment targets (41).

## 7 Role of lactylation in ICC

Intrahepatic cholangiocarcinoma (ICC) is the second most prevalent type of liver cancer. Ten percent or so of cholangiocarcinomas are ICC. It results from the peripheral bile channels of the liver parenchyma that are located close to secondary biliary radicals. Most ICCs are histologically classified as adenocarcinoma. With a median lifespan of less than three years, only 15% of individuals have a condition that can be treated at presentation. The deadly cancer known as intrahepatic cholangiocarcinoma (iCCA) is becoming increasingly common worldwide. The tumor-node-metastasis (TNM) staging method is widely used. However, its shortcomings in terms of patient prognosis prediction require the development of additional genetic markers (34, 42).

Tumor microenvironment (TME) factors, such as lactate metabolism, play a major role in immune modulation and tumor growth. A group of scientists led by Chen Sang, Li Yan, Jian Lin, Youpei Lin, Qiang Gao, and Xia Shen conducted a study in March 2024 that effectively established a six-gene lactate metabolism-related (LMRG) prognostic signature for prognosticating patients with intrahepatic cholangiocarcinoma (iCCA). They wanted to use lactate metabolism-related genes (LMRGs) to create a prognostic signature. The study provides a thorough examination of the relationship between intrahepatic cholangiocarcinoma (iCCA) and lactate metabolism-related genes (LMRGs). The important findings (34, 42)are: 1.Identification of LMRGs: Six important LMRGs that are substantially correlated with the prognosis of patients with iCCA were identified using sophisticated bioinformatics and experimental techniques. 2.Development of a Prognostic Model: Using LMRG scores as a basis, a prognostic model was created to identify patients at high and low risk of unfavorable outcomes. A worse prognosis, a higher prevalence of KRAS and TP53 mutations, and a weakened immune system, which mostly affects natural killer (NK) cells, were all linked to higher LMRG scores. 3.Therapeutic Implications: This study showed that one of the discovered LMRGs, lactate dehydrogenase A (LDHA), may be knocked down to suppress tumor development *in vivo* in mouse models, as well as tumor proliferation and migration *in vitro*. This suggests that novel treatment approaches for iCCA may be possible by focusing on lactate metabolism. 4.The LMRG high-score group had modified immune cell infiltration, suggesting an immunosuppressive tumor microenvironment (TME). This has an impact on the tumor microenvironment. This includes decreased function and infiltration of NK cells, which are essential for antitumor immunity. 5.This study demonstrates the importance of lactate metabolism in determining the immunological environment and course of iCCA, laying the groundwork for further research and possible therapeutic uses focusing on metabolic pathways for cancer treatment.

The influence of lactate metabolism on tumor growth and immunological interactions within the tumor microenvironment was highlighted, shedding insight into the understudied function of lactate metabolism in iCCA. The Boruta algorithm and univariate Cox regression were used to identify important lactate metabolism-related genes (LMRGs) and build a predictive model. Both *in vitro* and *in vivo* validations of this model demonstrated a relationship between high LMRG scores and unfavorable patient outcomes, as well as novel potential targets for treatment. They examined the role lactate metabolism plays in intrahepatic cholangiocarcinoma (iCCA), a liver cancer with a low prognosis and increasing prevalence. Six important genes linked to lactate metabolism were identified by researchers, who used these genes to build a predictive model that uses gene expression scores to predict patient outcomes. High scores were associated with a worse prognosis, more TP53 and KRAS gene alterations, and decreased natural killer (NK) cell activity, suggesting a weakened immune response. By modifying lactate metabolism, this model contributes to a better understanding of the tumor microenvironment and provides possible novel targets for treatment, with the goal of improving the treatment and prognosis of patients with intractable kidney cancer. For further understanding, refer to **Table 5**.

**Table 5:** Simplifies and clarifies the complex data by providing a concise summary of the study’s key findings, methodologies, and potential therapeutic implications of the identified LMRGs.

Study Objective	To discover and confirm a predictive marker for intrahepatic cholangiocarcinoma (iCCA) based on genes associated to lactate metabolism (LMRGs).
Methods Used	Boruta algorithm, *in vitro* and *in vivo* trials, and univariate Cox regression analysis
Key LMRGs Identified	LDHA, ATAD3A, ETHE1, LARS2, SLC13A5, SLC25A19
Key Findings	A bad prognosis is correlated with high LMRG scores.More TP53 and KRAS mutations are linked to higher LMRG scores.NK cell function was compromised in the group with a high LMRG score.(br>- Tumor development and iCCA cell proliferation were suppressed by LDHA knockdown.
- Biological Role of LDHA	Tumor cell migration and proliferation were inhibited by LDHA downregulation, suggesting that it may be a useful target for therapy.
Impact on Immune System	Tumor microenvironment immunosuppression is influenced by altered immune cell infiltration, particularly decreased NK cell function.
Conclusion	Based on LMRGs, the study created a trustworthy predictive model for iCCA and offered fresh perspectives on the metabolic control of tumor growth and immune evasion. Additionally, potential treatment targets for iCCA were found.

## 8 Role of lactate in colorectal cancer

One of the main causes of cancer-related mortality worldwide is colorectal cancer (CRC), and one of the characteristics of cancer cells is metabolic reprogramming, namely, aerobic glycolysis (the Warburg effect). Recent research has identified lysine lactylation as a crucial post-translational modification (PTM) that influences gene expression and metabolism, among other biological processes, ultimately affecting the development of cancer. Metabolic reprogramming has attracted interest because of its possible effects on tumor growth and metastasis, specifically on lactate function in colorectal cancer. To provide new opportunities for targeted therapeutics, recent research has focused on understanding how lactate metabolism influences the tumor microenvironment, cancer cell survival, and metastasis. There are four studies conducted on this topic, summarized in the **Table 6** (43–46), where the aims, results, approaches, and outcomes of each study are clearly compared, highlighting the various yet interconnected roles that lactate metabolism may play in the development and treatment of colorectal cancer.

**Table 6:** Highlights the diverse yet interconnected roles that lactate metabolism may play in the development and management of colorectal cancer (43–46).

No	Title	Main Focus	Key Findings
1	Exploring the glycolytic cross-talk genes between inflammatory bowel disease and colorectal cancer	Identification of cross-talk genes between IBD and CRC that affect glycolysis and CRC prognosis	Identified P4HA1 and PMM2 as glycolytic cross-talk genes impacting tumor microenvironment and immune response
2	GPR37 promotes colorectal cancer liver metastases by enhancing the glycolysis and histone lactylation via Hippo pathway	Role of GPR37 in promoting CRC liver metastasis via enhanced glycolysis and histone lactylation	GPR37 enhances CRC metastasis and glycolysis, promoting histone lactylation and upregulating genes involved in tumor progression
3	Role of PFKP lactylation in regulating glycolysis and cancer metabolism in colorectal cancer	Impact of PFKP lactylation on glycolysis and its regulatory role in CRC metabolism	Demonstrated that lactylation of PFKP reduces its activity, suggesting a feedback mechanism that could influence metabolic
4	Tumor-derived lactate promotes resistance to bevacizumab treatment by facilitating autophagy enhancer protein RUBCNL expression through histone H3 lysine lactylation	How tumor-derived lactate influences resistance to bevacizumab by affecting autophagy and histone lactylation	Found that lactate promotes autophagy and resistance to treatment through lactylation of histone H3, impacting gene expression related to survival

## 9 Role of lactylation in gastric cancer

Globally, gastric cancer remains the primary cause of cancer-related deaths, necessitating the development of innovative diagnostic and treatment strategies. Lactylation is a histone modification that was recently found to be produced from lactate. It has been linked to several processes associated with cancer including immune evasion, metastasis, and proliferation. Histone lactylation is an intriguing component of cancer metabolism that has drawn the attention of researchers in the ongoing fight against gastric cancer. Lactate is a consequence of the aggressive metabolism that cancer cells frequently display, and this novel epigenetic change has opened new avenues for studying and treating this debilitating disease. A comprehensive investigation into the proteome of stomach cancer cells was conducted to map lactylation hotspots, this study identified 2,375 lactylation sites across 1,014 proteins, many of which are crucial for essential biological functions, such as RNA splicing (47). The findings extended beyond mere categorization, revealing a significant link between higher lactylation levels and poorer patient prognoses in stomach cancer. This suggests that elevated lactate levels could serve as a potential biomarker for assessing disease severity (47). Building on previous findings, a clinical study developed a model based on lactylation to predict patient prognosis in stomach cancer, this model also assessed tumor infiltration by immune cells and predicted the tumor’s response to immunotherapy. The study found that tumors with higher lactylation scores exhibited reduced effectiveness of immune checkpoint inhibitors, therapies designed to reinvigorate the immune response against cancer. These tumors were more adept at evading immune surveillance, highlighting the potential role of lactylation in influencing therapeutic outcomes (48). The salient features and conclusions of the two studies on lactylation in gastric cancer are summarized in **Table 7**.

**Table 7:** Provides a clear overview of how each study contributes to our understanding of lactylation’s role in the progression of gastric cancer and its potential as a therapeutic target by contrasting the distinctive and overlapping aspects of the two investigations (47, 48).

Feature	Study 1: Identification of Lysine-Lactylated Substrates in Gastric Cancer Cells	Study 2: Lactylation-Related Model to Predict Prognosis and Immunotherapy Response
Focus	Analyzing the relationship between the prognosis of the disease and the lactylated protein profile in gastric cancer	Creating a model linked to lactylation in order to assess genetic instability, immunotherapy response, and immunological infiltration.
Key Findings	2375 lactylation sites were found in 1014 different proteins. Poor patient outcomes were connected with high levels of lactylation.	Genetic instability, immunological cell infiltration, and a poor response to immunotherapy are all correlated with lactylation scores.
Methods	Immunohistochemistry, survival analysis, and proteomic analysis	Survival analysis, cluster analysis, and bioinformatics
Significance	Shown that lactylation may be a useful prognostic indicator for gastric cancer.	Demonstrated that the likelihood of immune evasion and the efficacy of immunotherapy may be predicted by lactylation levels.
Implications for Therapy	Proposed that addressing lactylation might enhance treatment approaches and prognosis.	Suggested possible treatment targets to control immune evasion in gastric cancer and improve the effectiveness of immunotherapy.
Clinical Relevance	Facilitated the development of lactylation as a biomarker for the prognosis of stomach cancer.	Created a model that predicts how immunotherapy will work, which will help with tailored treatment plans for stomach cancer.

Lactylation provides valuable insights into the molecular dynamics of stomach cancer, with significant implications for treatment and prognosis. Future therapies may focus on targeting lactylation pathways, particularly in immunotherapeutic settings, to improve patient outcomes. Further investigation into the mechanisms underlying lactylation could identify specific enhancers or inhibitors with therapeutic potential. Additionally, clinical trials are needed to validate the proposed prognostic and therapeutic models (47, 48).

## 10 Role of lactylation in pancreatic cancer

Since cancer remains a major global health concern, understanding its molecular origins is essential for improving treatment outcomes. Recent studies have revealed that lactylation, a post-translational alteration, plays a significant role in the metabolic and epigenetic landscape of cancer, influencing the course of the disease and effectiveness of treatment.

The aggressive nature, late detection, and resistance to standard therapy of pancreatic adenocarcinoma contribute significantly to its continued status as one of the deadliest cancer types. It is crucial to develop new strategies for understanding and combating the metabolic pathways that pancreatic cancer cells use to survive. A crucial role of lactate addition to protein lysine residues, a newly identified post-translational modification, has been identified in the metabolic reprogramming of cancer cells. **Table 8** highlights the unique contributions of each study in advancing our understanding of lactylation’s role in cancer biology. It also outlines the implications for therapeutic strategies and suggests potential avenues for future research (49, 50).

**Table 8:** Offers an organized comparison, illustrating the distinct ways in which each study advances our understanding of lactylation’s role in cancer biology and highlights implications for treatment approaches and future research directions (49, 50).

Study Aspect	Huang et al. (2024)	Wu et al. (2024)
Title	Under glucose deprivation, pancreatic cancer cells are more likely to survive when L-lactate increases NMNAT1 lactylation and maintains the nuclear NAD+ salvage pathway.	A pan-cancer multi-omics investigation of lactylation genes linked to the development of cancer and the tumor microenvironment.
Focus of Study	Examines the role that L-lactate plays in pancreatic adenocarcinoma cells’ ability to survive when glucose is scarce.	Investigates how lactylation genes function in a variety of malignancies and how they affect the tumor microenvironment and immune response.
Key Mechanism Studied	L-lactate lactylates NMNAT1 to increase its activity in the NAD+ salvage pathway.	Comprehensive examination of the expression and mutations of lactylation genes, evaluating their relationship to the results of immunotherapy and the dynamics of the tumor microenvironment.
Methodologies	Enzyme activity assessments, glucose-deprived cell culture procedures, and biochemical tests	Clinical data correlation coupled with multi-omics data analysis, including transcriptomics and genomics.
Key Findings	L-lactate-induced NMNAT1 lactylation, mediated by the NMNAT1 gene, increases the effectiveness of the NAD+ salvage pathway, which is essential under nutritional stress and enhances cell viability.	Lactylation genes, including LDHA, GLS1, and OGDH, are associated with poor immunotherapy results, are increased in malignancies, and contribute to the immunosuppressive tumor microenvironment.
Clinical Implications	Identifies possible therapeutic targets for lactate metabolism disruption to prevent cancer cells from using their metabolic stress defense systems.	Demonstrates how lactylation pathways may be targeted to increase the effectiveness of immunotherapy and offers biomarkers for cancer prediction and treatment options.
Potential for Future Research	Additional research on lactate metabolism inhibitors as potential treatments for pancreatic cancer.	Thorough analysis of lactylation as a diagnostic and therapeutic target for different forms of cancer.

## 11 Role of lactylation in prostatic cancer

Prostate cancer continues to be the primary cause of cancer-related deaths in men; therefore, new methods for diagnosis and treatment are required. Recent studies have identified protein lactylation as a critical post-translational alteration that affects the course of cancer and effectiveness of therapy. This study examined the results of three studies that used gene signatures related to lactylation to predict disease-free survival and evaluate the efficacy of prostate cancer treatments. We summarized the new findings based on three important studies on the involvement of lactylation in prostate cancer. Through machine learning models, these studies investigated the efficacy of lactylation-related gene signatures in predicting disease-free survival and treatment response. The knowledge gained from these research projects might be used in clinical settings to improve the prognosis of patients with prostate cancer and to tailor treatment plans. **Table 9** highlights and contrasts the main findings of the three studies on lactylation in prostate cancer (51, 52).

**Table 9:** Provides a systematic comparison, highlighting the unique contributions and conclusions of each study, and illustrating how lactylation impacts the prognosis and treatment options for prostate cancer (51, 52).

Study Aspect	Pan et al. (2023) - Study 1	Pan et al. (2023) - Study 2	Zhang et al. (2023)
Title	Create a lactylation-related gene prognostic model to accurately forecast prostate cancer treatment response and disease-free survival.	Identifying Key Prostate Cancer Biomarkers Linked to Lactylation.	Gene Signatures Associated with Lactylation as Predictors of Disease-Free Survival and Therapy Response in Prostate Cancer
Focus	Development of a Predictive Model Using Lactylation-Linked Genes to Forecast Treatment Response and Disease-Free Survival.	Exploring the Relationship Between Prostate Cancer Prognosis and Lactylation Biomarkers.	Analyzing Lactylation-Related Gene Profiles to Predict Treatment Response and Disease-Free Survival.
Key Findings	Identified five key lactylation-related genes (LRGs) and developed a prognostic model that accurately predicts treatment response and survival.	Emphasized the role of specific lactylation-related genes in immune cell invasion and the tumor microenvironment.	Demonstrated that lactylation-related gene signatures have significant predictive value in stratifying patients into risk categories.
Methodologies	Developed the prognostic model using Lasso Cox regression analysis and machine learning techniques.	Utilized research on immune cell infiltration and differential gene expression analysis.	Utilized a comprehensive multi-omics approach to analyze the expression and effects of lactylation-associated genes.
Practical Consequences	Demonstrates the model’s potential for use in clinical settings to forecast patient outcomes and personalize treatment regimens.	Suggests that lactylation may be the target of therapeutic intervention, improving patient classification for treatment.	Focuses on the use of lactylation-related gene profiles as useful indicators for treatment planning and patient prognosis.
Possibilities for Additional Research	Conducted additional clinical trial validation of the prognostic model and explored the underlying processes influencing response to gene therapy.	A comprehensive analysis of the effects of lactylation on immune evasion and the tumor microenvironment in prostate cancer.	Expanding the use of this research to create more personalized and accurate treatment plans for prostate cancer patients

## 12 Role of lactylation in renal and bladder cancer

The development of resistance to therapy in bladder and renal malignancies is attributed to different epigenetic landscapes and metabolic reprogramming. Lactylation occurs when lactate is added to lysine residues on histones. It has been identified as a key alteration that combines epigenetic modifications with metabolic disruptions to affect tumor behavior in the two forms of cancer.

### 12.1 Role of lactylation in renal cell carcinoma

Some metabolic changes that are specific to ccRCC contribute to its development. Recent research has focused on a unique epigenetic alteration known as histone lactylation and its involvement in controlling various metabolic pathways. Renal cell carcinoma (ccRCC) is a common type of kidney cancer that is characterized by changes in metabolism and resistance to conventional cancer treatments. The function of epigenetic modifications, namely histone lactylation, in influencing these metabolic alterations and propelling the spread of cancer has been gradually revealed by recent studies. Targeting metabolic and epigenetic pathways in ccRCC has the potential to yield considerable insights into these processes, as demonstrated by some studies. An important epigenetic alteration in ccRCC is histone lactylation, which is closely linked to the metabolic structure of the tumor. The studies by Liu et al. and Zhang et al. expand our knowledge of the metabolic and epigenetic landscape of colorectal cancer (ccRCC), offering a viable basis for the creation of novel treatment approaches that target these interrelated processes, highlighting the critical role of lactylation in ccRCC metabolic reprogramming and epigenetic control. Histone lactylation not only meets the energy and biosynthetic needs of tumor cells by modifying important metabolic enzymes, such as LDHA, but also offers a means for the survival of oncogenic signaling pathways. These results implied that preventing these interactions could be beneficial from a therapeutic standpoint when treating ccRCC. Collectively, these investigations greatly advance our knowledge of how changes in metabolic pathways, resulting from protein modifications such as lactylation, are essential for the development of cancer and its resistance to treatment. By focusing on the metabolic mechanisms of ccRCC, this information creates new treatment opportunities (53).

### 12.2 The impact of CircXRN2 and histone lactylation on bladder cancer progression

Bladder cancer ranks among the top ten most common malignancies worldwide, with a notably high recurrence and fatality rate. Recent investigations have explored the interaction between epigenetic modifications, such as histone lactylation, and noncoding RNAs, particularly circXRN2, which play a critical role in the etiology and progression of bladder cancer. One study specifically examines the function of circXRN2, revealing its significant downregulation in cancerous tissues and cell lines, suggesting a potential tumor-suppressor role. Furthermore, evidence indicates that circXRN2 may inhibit tumor growth by regulating histone lactylation and activating the Hippo signaling pathway, thereby offering new insights into therapeutic strategies (54). Lactylation plays a crucial role as an epigenetic mediator between metabolic changes and control of gene expression in bladder and renal malignancies. The increasing recognition of its function presents promising prospects for innovative treatment approaches that target the metabolic and epigenetic underpinnings of these malignancies. **Table 10** highlights the key findings from the three studies examining the relationship between lactylation and bladder and renal malignancies (53–55).

**Table 10:** Analyzes and contrasts the findings and techniques of three key studies, illustrating how lactylation affects renal and bladder cancer pathogenesis and offering potential avenues for therapeutic intervention (53–55).

Study Aspect	Yang et al. (2022) - Renal Cancer	Liu et al. (2024) - Renal Cancer	Xie et al. (2023) - Bladder Cance
Title	Clear cell renal cell carcinoma progression is driven by a positive feedback loop between PDGFRβ signaling and inactive VHL-triggered histone lactylation.	Through its facilitation of LDHA phosphorylation, FKBP10 enhances the advancement of clear cell renal cell carcinoma and controls susceptibility to HIF2α inhibition.	In human bladder cancer, CircXRN2 inhibits the growth of tumors caused by histone lactylation by turning on the Hippo pathway.
Focus of Study	Analyzes how enhanced histone lactylation caused by inactive von Hippel-Lindau (VHL) may accelerate the development of renal cancer by activating the PDGFRβ signal.	Analyzes the ways in which FKBP10 impacts the response to HIF2α inhibitors and the development of renal cancer by increasing histone lactylation and enhancing LDHA phosphorylation.	Analyzes how the Hippo pathway and modification of histone lactylation are two ways that the circular RNA circXRN2 controls the growth of tumors in bladder cancer.
Key Mechanisms	Inactive VHL triggers histone lactylation, which amplifies PDGFRβ signaling in a feedback loop and accelerates the growth of tumors.	Through binding to LDHA, FKBP10 enhances phosphorylation of the molecule, which raises histone lactylation. This leads to an overactive Warburg effect and affects the susceptibility of renal cancer to HIF2α inhibitors.	Through inhibiting SPOP-mediated degradation of LATS1, circXRN2 increases its stability. This activates the Hippo signaling pathway, which in turn decreases H3K18 lactylation and lowers LCN2 expression in bladder cancer.
Methodologies	Used *in vivo* models, genomic analysis, and immunohistochemistry to investigate the connection between histone lactylation, VHL status, and the advancement of ccRCC.	Used *in vivo* studies, bioinformatics, and molecular biology methods to investigate the relationship between FKBP10 and LDHA and how it affects the course of renal cancer as well as treatment sensitivity.	Explored the involvement of circXRN2 and histone lactylation in bladder cancer using RNA immunoprecipitation, overexpression and knockdown experiments, Seahorse metabolic analysis, CUT&Tag, and ChIP tests.
Clinical Consequences	Suggests that one possible treatment approach for ccRCC is to target the VHL-PDGFRβ-lactylation axis, especially by breaking the feedback loop that encourages tumor development.	Identifies possible targets for treatment in the interactions between FKBP10 and LDHA to prevent metabolic reprogramming in renal cancer and increase the effectiveness of HIF2α inhibitors.	Demonstrates how altering metabolic and epigenetic pathways to increase or imitate the activity of circXRN2 may be a unique therapeutic approach for treating bladder cancer.
Potential for Future Research	Further investigation into additional signaling pathways impacted by histone lactylation may provide additional targets for therapeutic intervention in renal cancer.	Extensive research on the wider consequences of FKBP10 interactions in renal and maybe other malignancies may shed light on both generic and targeted anti-cancer treatments.	Future research might examine the wider ramifications of Hippo pathway activation in different cancer types, as well as the therapeutic potential of circXRN2 overexpression or imitation in clinical situations.

The findings show that lactylation influences tumor development and treatment response by serving as a link between metabolic and epigenetic changes in cancer cells. Recent research indicates that lactylation is an important factor in cancer biology and may be a target for cutting-edge cancer treatments, particularly for difficult-to-treat diseases, such as bladder and kidney tumors.

## 13 Role of lactylation in lung cancer

Globally, lung cancer remains the leading cause of cancer-related deaths, particularly in non-small cell lung cancer (NSCLC) and lung adenocarcinoma (LUAD). Tumor growth and treatment resistance are significantly influenced by metabolic reprogramming in cancer cells, including changes in lactylation. Our understanding of how lactylation affects metabolic pathways and gene expression in lung cancer has advanced significantly, paving the way for new treatment options. Recent studies have examined crucial aspects of metabolic control in LUAD and NSCLC, focusing particularly on lactylation dynamics. The complex relationship between lactylation and lung cancer metabolism has been emphasized, showing that lactylation can directly influence the expression of metabolic genes, thereby altering cellular energy processes. Furthermore, the connection between lactylation and specific transport proteins that affect cancer cell survival and their interactions with the surrounding environment has been expanded, enhancing our understanding of lactylation’s role. These findings suggest that targeting lactylation and its downstream effects may provide innovative approaches for lung cancer treatment, particularly in improving the efficacy of existing therapies or developing new ones. Emphasizing lactylation-induced metabolic reprogramming may yield novel therapeutic strategies for lung cancer management, especially since modifying lactylation could change the metabolism of cancer cells and enhance their sensitivity to current treatments (56, 57). Clinical trials exploring lactylation inhibitors or modulators may also provide insights into their therapeutic potential. Future research should explore the broader implications of lactylation in other cancer types and the detailed mechanisms by which lactylation influences metabolic pathways.

## 14 Histone lactylation in ocular melanoma

Histone lactylation is a post-translational modification that arises from lactate and is increasingly being understood to play a role in controlling the expression of genes linked to stress responses and cellular metabolism. Its effects on cancer, especially ocular melanoma, a disease with poor prognosis and a reputation for aggression, have been the subject of recent studies that shed light on the role of histone lactylation in ocular melanoma and suggest that targeting this modification may be a promising strategy for cancer therapy. Future research should explore the broader applications of these findings across different cancers and investigate combination therapies. Both studies noted that elevated histone lactylation is linked to worse patient outcomes, emphasizing its potential as a prognostic marker. These findings highlight the dual roles of metabolic processes and epigenetic modifications in cancer pathology. **Table 11** provides a comprehensive overview of how each study contributes to our understanding of the role of histone lactylation in the development of ocular melanoma by contrasting the unique and overlapping aspects of the two studies (58, 59).

**Table 11:** Provides a clear illustration of how each study contributes to our understanding of the role of histone lactylation in the development of ocular melanoma by comparing the distinctive and overlapping aspects of the two studies (58, 59).

Aspect	Study 1: ALKBH3 and Histone Lactylation	Study 2: YTHDF2 and Histone Lactylation
Focus	Role of histone lactylation-mediated ALKBH3	Histone lactylation plays a role in YTHDF2.
Key Findings	Histone lactylation disrupts tumor-suppressive nuclear bodies, increasing the expression of ALKBH3, and accelerating the development of melanoma.	Through destroying tumor-suppressor mRNAs, histone lactylation promotes the production of YTHDF2, which speeds up the process of carcinogenesis.
Methods	*In vitro* and *in vivo* functional tests; ChIP-seq; RNA-seq; immunofluorescence	Functional tests, such as ChIP-seq, MeRIP-seq, and RNA-seq, both *in vivo* and *in vitro*
Importance pertaining to Cancer Progress	Reveals a molecular relationship in cancer cells between transcriptional activation, histone modification, and metabolic stress.	Connects the dots between RNA and histone alterations, exposing a new regulatory axis in cancer
Therapeutic Implications	Targeting ALKBH3 or changing histone lactylation may provide melanoma patients with additional therapy alternatives.	YTHDF2 is proposed as a possible therapeutic target, with implications for controlling ocular melanoma via altering the lactylation of histones.
Prognostic Value	Elevated levels of histone lactylation have been linked to a worse prognosis and more aggressive malignancy.	Increased histone lactylation may be a prognostic marker as it is linked to poorer outcomes.
Clinical Relevance	Emphasizes how crucial it is to comprehend epigenetic modifications in cancer treatment plans.	Highlights the necessity of treatments that address both RNA and epigenetic changes in order to effectively control cancer.

## 15 Histone lactylation in cancer progression and immunotherapy efficacy

Recent studies have revealed that histone lactylation acts as a crucial link between cellular metabolism and epigenetic modifications, influencing various biological processes, including immune response regulation and cancer development. These findings suggest potential therapeutic benefits of targeting histone lactylation in multiple cancer types. For instance, modulating lactylation and glucose metabolism may enhance bone formation therapies for malignancies associated with osteoblasts. Additionally, interrupting lactate-driven histone modifications in hematological malignancies, such as acute myeloid leukemia (AML), could improve the efficacy of immunotherapies. However, further investigation is needed to explore histone lactylation across different malignancies and its broader implications in diseases characterized by immunological dysregulation and altered metabolism. **Table 12** provides a summary and comparison of the main findings from both investigations, highlighting their distinct yet complementary areas of interest regarding the role of histone lactylation in immunological modulation and cellular differentiation (60, 61).

**Table 12:** This table summarizes and compares the key findings from both investigations, emphasizing their distinct and complementary areas of focus on the role of histone lactylation in immunological modulation and cellular differentiation (60, 61).

Feature	Study 1: Differentiation of Osteoblasts.	Study 2: Immunotherapy with AML.
Title	Lactate-induced histone lactylation by p300 promotes osteoblast differentiation.	Long, STAT5 promotes PD-L1 expression by facilitating histone lactylation to drive immunosuppression in acute myeloid leukemia, Signal transduction and targeted therapy.
Objective	To investigate the role of histone oxidation in the development of osteoblasts	To examine how PD-L1 expression and immune evasion in AML are influenced by STAT5-induced lactate production through histone lactylation.
Methods	ChIP-seq analysis of gene expression and cellular tests for osteoblast differentiation.	ChIP-seq analysis of gene expression, analytical metabolic tests, and assays for immune cell activity
Key Finding	Through p300, histone lactylation enhances the expression of bone development markers, thereby promoting osteoblast differentiation.	Through histone lactylation, STAT5-mediated lactate synthesis upregulates PD-L1 expression, aiding AML cells in evading the immune system.
Importance to Cancer	Suggests a biochemical link between osteoblast differentiation and potential interventions for bone health.	Demonstrates how leukemia involves a metabolic-immune checkpoint axis and how metabolic therapies could enhance the effectiveness of immunotherapy.
Approaches Used	Elevated glucose tests, genome study of osteoblast marker genes, and analysis of histone modifications.	Glycolytic profile analysis and assessment of PD-L1 expression assays for T-cell function.
Potential Benefits to Therapy	Therapies targeting bone production and health could be enhanced by modifying glucose metabolism and histone lactylation.	In AML, targeting histone lactylation and lactate production may improve the response to immune checkpoint inhibitors.

## 16 Limitations

The significance of lactylation in cancer is the main topic of this review, which also highlights how it affects tumor biology and possible treatment options. Many molecular pathways remain unknown, and the area of lactylation research is still in its infancy. The research cited here is constrained by its experimental models, which frequently use animal models or *in vitro* settings that might not accurately capture the complexities of human cancers. Additionally, although many studies offer encouraging insights, they are frequently limited to particular cancer types, and more research is needed to determine their wider application across various tumor situations. Our knowledge of lactylation’s entire role in gene regulation is also limited by its understudied interaction with other post-translational modifications.

## 17 Conclusion

Immune regulation, metabolic reprogramming, and tumor growth are significantly affected by lactylation, a new epigenetic alteration. The addition of lactate to the lysine residues of histone proteins adds a new level of complexity to the study of cancer by tying metabolic changes to gene expression. The translational potential of the lactylation pathway targeting in cancer treatment is highlighted in this study, providing novel therapeutic intervention routes. The molecular processes of lactylation and their significance in different cancer types, especially in relation to immune evasion and resistance to existing treatments, require further investigation.
